# Diseases of Pet Guinea Pigs (*Cavia porcellus*) Presented to a Specialist Exotic-Animal Clinic: A Retrospective Study

**DOI:** 10.3390/ani16193026

**Published:** 2026-09-25

**Authors:** Adrienn Aliza Keszeli, Tamás Sátorhelyi, Zsolt Lang, Nikoletta Hetényi

**Affiliations:** 1 Department of Animal Nutrition and Clinical Dietetics, Institute for Animal Breeding, Nutrition and Laboratory Animal Science, University of Veterinary Medicine, István u. 2., 1078 Budapest, Hungary; keszeli.adrienn.aliza@student.univet.hu; 2 Exo-Pet Veterinary Center, Hernád u. 40., 1078 Budapest, Hungary; satorhelyi@gmail.com; 3 Department of Biomathematics, University of Veterinary Medicine, István u. 2., 1078 Budapest, Hungary; lang.zsolt@univet.hu

**Keywords:** nutrition, disease, prevention, survey, health, malocclusion, neoplasia, urinary disease, ovarian cyst, prevention

## Abstract

This retrospective study describes the relative frequency of common diseases in pet Guinea pigs (*Cavia porcellus*, GP) presented to a specialist exotic-animal clinic (Exo-Pet Veterinary Center, Budapest, Hungary). Data from 600 GPs across 1768 visits were reviewed; 51.3% were male and 48.7% female. Malocclusion of the molars was the most common problem (27.5%), followed by urinary tract diseases (18.67%), reproductive tract diseases (16.2%), ectoparasites (16.5%), neoplasms (tumors), and tumor-like lesions (15.8%). We fitted multivariable logistic-regression models with coat type, age, and sex as predictors. Almost one-third of the intact females (32.40%) had ovarian cysts. Age was associated with the recorded incidence of several diseases, although diagnostic opportunity was generally greater in older animals. The incidence of infectious diseases (e.g., fungal disease) declined with age. Owners should be made aware of ectoparasites and fungal infections to prevent potential zoonotic disease. The odds of ovarian cysts, neoplasms (tumors) and tumor-like lesions, gastrointestinal, hepatobiliary and urinary disease increased with age; these associations remained after adjustment for the number of veterinary visits. Males are more likely to develop malocclusion of the molars and incisors. Regular, detailed dental examinations of GPs are important for both prevention and early diagnosis.

## Simple Summary

Guinea pigs (*Cavia porcellus*, GPs) are popular pets; however, husbandry and nutritional diseases are common in this species. Epidemiological data on the occurrence and distribution of diseases in pet GPs, particularly in Central and Eastern Europe, are limited. This retrospective study describes the relative frequency of the most common diseases among pet GPs presented to a specialist exotic-animal clinic. We also examine associations between the occurrence of certain diseases and the age, sex, and coat type of the GPs. Because the animals were drawn from a single specialist clinic rather than the general pet population, these findings may reflect clinic-based frequencies rather than population prevalence. Several diseases were more common in older GPs, and some health problems were more common in males. Some of the frequently recorded conditions (e.g., dental disease) have been linked to nutrition in previous work. Although we could not collect dietary information, these findings reinforce the general importance of owner education on appropriate diet and husbandry.

## 1. Introduction

Guinea pigs (*Cavia porcellus*, GPs) are popular household pets. Despite information on their husbandry and nutrition being readily available, nutrition and husbandry diseases are common in these animals [1]. As hindgut fermenters with continuously growing teeth, GPs require a high-fiber diet based on ad libitum hay, with only limited amounts of pelleted feed and fresh produce [2,3,4]. Among the diseases potentially influenced by nutrition, dental and gastrointestinal diseases are the most important [1,2]. The latter is mostly associated with diets low in fiber and/or high in starch; however, it may also have a genetic background. An imbalanced diet high in energy and low in fiber may also lead to obesity, glucose intolerance, dyslipidemia, nonalcoholic fatty liver disease, cardiac, and kidney disease [5,6]. In addition to nutritional factors, disease occurrence in GPs may also be influenced by age, sex, breed, and genetics. Previous studies have shown that older animals are more prone to neoplastic, dental, and metabolic disorders [7]. Some diseases (e.g., urinary tract and skin diseases) may occur more frequently in one sex [7,8,9]. However, a retrospective study using VetCompass data found no significant differences in longevity by sex, neutering status, or breed [10]. Few studies have examined the health of pet GPs, especially those based on veterinary databases [7,9,10,11,12,13,14]. Some surveys focused on the occurrence of certain diseases, such as obesity [15], ocular diseases [16], urolithiasis [17], skin diseases [8], and dental diseases [18], or on GP welfare [19,20].

Epidemiological data on the occurrence and distribution of diseases in pet GPs, particularly in Central and Eastern Europe, are limited. Thus, this study aimed to evaluate the health and most common diseases of pet GPs using a veterinary database. We investigated associations between the occurrence of certain diseases and the age, sex, and coat type (long- vs. short-coated breeds) of the GPs. Retrospective, clinic-based descriptions of disease in GPs remain relatively few. The most comprehensive to date are the Czech single-clinic study of Minarikova et al. (2016) [7] and the UK primary-care VetCompass study of O’Neill et al. (2024) [14]; comparable data from Hungarian and other regional specialist caseloads are lacking. This study adds a caseload from a Hungarian specialist exotic-animal clinic in which most animals underwent a detailed diagnostic work-up, including molar examination under anesthesia. Using the veterinary database enables us to identify conditions that are often underdiagnosed in owner questionnaires (e.g., malocclusion of the molars and ovarian cysts). The results could be useful to practicing veterinarians, especially for diseases that screening can prevent.

## 2. Materials and Methods

We collected data at Exo-Pet Veterinary Center Budapest, Hungary, one of the largest exotic animal clinics in the country. GPs attended the clinic between March 2015 and April 2025. Before sampling, we applied the selection criteria to all 776 database entries. We excluded animals listed in the database but with no entry in their medical records (*n* = 11). This can happen if a GP is registered in the database but did not attend an examination at the clinic. From the resulting population meeting the criteria (*n* = 765), we selected 600 individuals using simple random sampling without replacement, using a fixed random number seed. We manually reviewed the GP subset to identify the diagnosis. The record review period was 6 months. The observation period for each animal was defined as the interval from its first to its last recorded attendance. The individual GP served as the unit of analysis, rather than the veterinary visit or diagnosis record.

For each disease category, an animal was classified as affected if the condition was recorded at any point in its history and as unaffected otherwise; a diagnosis recorded at multiple visits (e.g., a recurring dental disorder) was counted only once, so each animal contributed a single observation to each model. Consequently, the reported frequencies represent the cumulative proportion of animals recorded with each condition over varying observation periods, not point prevalence.

We recorded the animals’ age, body weight, breed, sex, and neutering status. We also recorded the total number of veterinary visits and dental treatments (Table 1). All diagnoses were made by a veterinarian specializing in exotic pets. If the physical examination was insufficient to establish a diagnosis, additional tests were performed. This included radiography, ultrasound examination, laboratory testing, microscopic examination, cytology, and histology. All dental examinations were carried out on anaesthetized animals. We separated diagnoses into presumptive and confirmatory. Clinical history, clinical signs, and physical examination supported presumptive diagnoses. Confirmatory diagnoses were established by using reference-standard tests (Supplementary Table S1). Both presumptive and confirmatory diagnoses were included in the statistical tests. We assigned categories pragmatically based on the presenting organ system. Conditions that can be classified into more than one category were recorded only once. Neoplasms (tumors) and tumor-like lesions were listed in a separate category, rather than under the relevant organ system (mammary neoplasia, uterine neoplasia, and tumor-forming on the thyroid gland are listed in this category, not as an organ specific disorder). This retrospective study used only anonymized clinical records from client-owned GPs that were examined and treated as part of routine veterinary care; no animal underwent any procedure for the study. Thus, ethical approval was not required. We could not collect information on feeding and housing conditions.

Odds ratios were exponentiated coefficients, with Wald 95% confidence intervals estimated by maximum likelihood. For each predictor, the null hypothesis that its coefficient equals zero (odds ratio = 1) was tested using a two-sided Wald test. The 95% confidence interval for each odds ratio was derived from the corresponding Wald interval. Then, each disease model was refitted (age per year, sex and coat type) with the number of veterinary visits added as a covariate, so that the age term was estimated at a comparable level of examination opportunity; sex and coat type are time-invariant and were retained only to keep the specification identical to the primary model. For each of the 13 diseases (malocclusion of the molars, malocclusion of the incisors, dental abscesses, obesity, gastrointestinal disease, ovarian cyst, neoplasms (tumors) and tumor-like lesions, respiratory disease, urinary disease, fungal disease, ectoparasites, ocular disease and hepatobiliary disease), a multivariable logistic regression was fitted with coat type (long vs. short coat), age (per year), and sex (male vs. female), using short and female as references. Age was adjusted to the date of diagnosis (first entry in the database). Odds ratios were not estimated for diseases with fewer than 20 cases, because three predictors cannot be supported reliably in such small strata. This excluded arthritis and cardiac disease. *p*-values < 0.05 were considered significant. The relationship between the number of different diagnoses recorded and the total number of veterinary visits was assessed using Spearman’s rank correlation coefficient. The model was refitted with 13 diseases (age per year, sex, and coat type), incorporating the total number of veterinary visits as a covariate. Sex and coat type, being time-invariant, were retained to maintain consistency with the primary model specification. We performed statistical analyses using R 4.5.1 statistical software [21] (R Core Team, 2025).

## 3. Results

### 3.1. Breed, Age and Sex

We recorded data on 600 GPs, representing 1768 veterinary visits. Table 1 shows the results of the recorded parameters. GPs examined on more occasions were more likely to have additional conditions recorded (Spearman’s *p* = 0.58, 95% CI 0.52–0.63). The total number of visits is, partly, a consequence of the disease itself. At the time of the last veterinary visit, almost half of the GPs were ≤3 years old (45.8%, *n* = 275), and 54.2% (*n* = 325) were older. Only 17.3% (*n* = 104) of the GPs were neutered. In total, 13 different registered breeds were represented (Figure 1).

### 3.2. Diseases of the Guinea Pigs

Seventy-seven animals (12.8%) had no medical disorders. These GPs arrived for a general medical check-up. This included a preventive dental check-up, nail trimming, and a general medical check-up or the animal was accompanied by another GP owned by the same person. The minority of GPs (19.0%, *n* = 114) required intensive hospital care. Due to serious health conditions, from an animal welfare perspective and on prognostic grounds, clinical euthanasia was performed in 8.7% (*n* = 52) of cases at the clinic. Euthanasia was carried out in accordance with the protocol of Smith (2022) [22].

Figure 2 shows the relative frequency of diseases diagnosed in GPs. Molar malocclusion was the most frequent disorder (27.5%, *n* = 165). Incisor malocclusion occurred in 13.2% of the animals (*n* = 79), while dental abscesses were found in 29 individuals (4.8%). Both molar and incisor malocclusions occurred in 66 cases (11.0%), and 13 GPs (2.2%) had both types of malocclusions with abscesses. The most frequently affected molars were the right maxillary molar 2 (*n* = 78) and right maxillary molar 3 (*n* = 78). These were followed by the right maxillary molar 1 (*n* = 76) and premolar (*n* = 72). The least affected tooth was the left mandibular molar 2 (*n* = 50) (Table 2).

The second most common condition was urinary tract disease (*n* = 112, 18.7%; Figure 3). Ectoparasites affected 16.5% (*n* = 99) of the GPs. Specifically, this included scabies (*n* = 45, 7.5%), lice infestation (*n* = 38, 6.3%), and unspecified ectoparasites (*n* = 16, 2.7%).

Neoplasms (tumors) and tumor-like lesions were also relatively common, with 95 cases (15.8%). Specifically, this was atheroma (3.7%, *n* = 22), mammary neoplasia (3.0%, *n* = 18), uterine neoplasia (1.7%, *n* = 10), lipoma (1.7%, *n* = 10), tumor-forming on the thyroid gland (1.0%, *n* = 6), and other tumor-forming conditions (4.8%, *n* = 29).

Reproductive tract disorders were diagnosed in 16.2% of GPs (*n* = 97). Ovarian cysts dominated (*n* = 93). Almost one-third of the 287 intact females (*n* = 93, 32.4%) had ovarian cysts. Twenty-nine females were neutered after diagnosis. Male reproductive tract disorders (0.5%, *n* = 3; paraphimosis *n* = 2; testicular atrophy) and female reproductive tract disorders (0.2%, *n =* 1, vaginal prolapse) were not common.

Ocular diseases were observed in 10.8% (*n* = 65) of the animals (Figure 4), followed by respiratory diseases (*n* = 62, 10.3%; Figure 5). Forty-four GPs had gastrointestinal disorders (7.3%; Figure 6).

Fungal disease affected 33 GPs (5.5%), dominated by dermatophytosis (*n* = 25, 4.2%) and eight unspecified cases (1.3%). Cardiovascular diseases affected seven animals (*n* = 7; hypertrophic cardiomyopathy *n* = 4, cardiac failure *n* = 2, cardiomegaly *n* = 1).

Hepatobiliary disease affected twenty-five (4.2%) GPs, namely mostly hepatomegaly (*n* = 23) and two unspecified liver disorders. Traumatic injuries were not common (*n* = 21, 3.5%), including fracture of incisors (*n* = 4), sprain/bruise (*n* = 4), bites (*n* = 3), fall (*n* =2), fracture (*n* = 2), and other conditions (*n* = 4, claw injury, eye injury, auditory canal trauma, herniated disc).

Pododermatitis occurred in twenty (3.3%) GPs. Skeletal diseases (*n* = 13, 2.2%) included mandibular osteolysis (*n* = 6), osteoporosis (*n* = 3), spinal deformity (*n* = 2), osteodystrophy (*n* = 1), and hypocalcemia osteopathy (*n* = 1). Fourteen (2.3%) GPs had arthrosis/arthritis, followed by otitis (*n* =10, 1.7%).

Eleven GPs had thyroid diseases (1.8%), and all of these were hyperthyroidism. The mean body weight of the GPs was 0.9 ± 0.1 kg; sixty (10.0%) animals were obese. The least common disorders were overgrown nails (*n* = 4, 0.7%), hernia (*n* = 3, 0.5%), toxicosis (*n* = 3, 0.5%), and coagulopathy (*n* = 2, 0.3%). Figure 7 shows the most frequently observed clinical signs.

Table 3 shows the 95% confidence intervals for the age (in years) of the animals affected by that condition. Table 4 shows the adjusted odds ratios with 95% confidence intervals for all 13 diseases. Males had higher odds of molar malocclusion and incisor malocclusion. Age raised the odds of ovarian cyst, dental disease, neoplasms (tumors) and tumor-like lesions, hepatobiliary, urinary, gastrointestinal disease, and respiratory disease, but lowered fungal disease and ectoparasites. Following adjustment for the number of veterinary visits, the associations between increasing age and ovarian cyst, neoplasia, urinary disease, and gastrointestinal disease remained significant. Similarly, the inverse associations for ectoparasites and fungal disease persisted. In contrast, age was not significantly associated with molar and incisor malocclusion, respiratory, or ocular disease.

## 4. Discussion

Epidemiological data on the health status and clinical signs of pet GPs remain limited, making it difficult to compare studies directly. The exact prevalence of many diseases likely varies with husbandry practices and study design. Therefore, before relying heavily on these population statistics, cross-check them against primary epidemiological data. To our knowledge, this is one of the first studies (except for [7]) in Central and Eastern Europe to present the spectrum of diseases affecting pet GPs based on a veterinary clinical database. The sex distribution was balanced (~50–50%), consistent with previous reports from clinical studies and surveys [7,9,14,15,18,20]. GPs at the time of the last veterinary examination were older than in other studies; however, comparison is difficult because they used different age ranges [7,9,14,15,18,23]. Neutering was rare (17.3%), close to the 12.0% reported by Olsen et al. (2025) [9], but considerably lower than the approximately 40% documented in various surveys ([19], 39.8%); ([20], 40.39%). Breed comparison is difficult, as several previous studies reported crossbreeds or unknown breeds. Among the purebreds, Abyssinians were most commonly recorded [9,14,19].

In terms of general health status, most GPs had a medical condition. This supports the retrospective findings of Minarikova et al. (2016), who reported that only 8.1% of 1000 GPs were healthy [7]. Similarly, O’Neill et al. (2024) [14] documented that in their cohort (*n* = 3785), only 13.13% had no medical records indicating any illness. In contrast, surveys relying on data provided by pet owners often show a much higher rate of good health (96.5% healthy GP) [9], highlighting a discrepancy between veterinary clinical diagnoses and owners’ perceptions.

Dental diseases represent a major health problem for GPs. This high relative frequency (45.3%) is consistent with findings from Minarikova et al. (2016, 36.6%) and Williams and Sullivan (2010, 45%) [7,16]. However, several studies reported much lower prevalence of dental diseases ([9], 8.1%); ([13], 14%); ([14], 8.1%); ([16], 6.7%); ([19], 4.9%); ([24], 23.4%). This may reflect methodological differences, as in the present study, all dental examinations were performed on anaesthetized animals using appropriate instruments. Oral examinations of awake animals and owner-completed questionnaires significantly underestimate the occurrence of dental disease. For example, Norman and Wills found that only 16.6% of GPs showed clinical signs suggestive of dental disease (e.g., epiphora or salivation) [18]. Male sex was associated with malocclusion of both the incisors and molars, which is consistent with the findings of O’Neill et al. [14]. Minarikova et al. (2016) reported odontogenic abscesses in 3.1%, similar to this survey (4.8%) [7]. The molars of the upper right jaw were the most commonly affected, while Jekl et al. [24] reported a predominance of molars from the lower jaw; the reason for this discrepancy is unclear and may relate to differences in diet, examination technique, or data collection. Diet affects dental health, as long-fiber feed promotes lateral chewing movements and occlusal wear. In contrast, abrasive pellets do not have such effects [11,12], although Norman and Wills [18] found no significant association with diet. Genetics and mineral balance factors also play a role in malocclusion development [11]. Because we could not obtain feeding data for the present study, we were unable to directly investigate this association.

Neoplasms (tumors) and tumor-like lesions were relatively frequent (15.8%), exceeding the reported frequencies [7,14]. Minarikova et al. (2016) reported skin tumors in 4.9%, mammary gland carcinoma in 1%, and gastrointestinal spindle cell sarcoma in 3 GPs [7]. In the survey by O’Neill et al. (2024), the incidence of abdominal mass was 1.3% and neoplasia was 3.9% [14]. Chen et al. (2024) collected samples from 20 GPs in which lipoma (6/20) was the most common, followed by an adenocarcinoma of the mammary gland (4/20), and collagenous hamartoma [25]. According to Greenacre (2004), the incidence of neoplasia, other than skin tumors, appears low among GPs [26]. The spectrum recorded here, dominated by cutaneous and mammary tumors and tumor-like lesions, is consistent with the limited case series available [7,25]. The higher incidence of these diseases in this survey may reflect the cohort’s older age, as odds increase with age [7]. In addition, the incidence of tumor diseases can be much higher in veterinary databases than in owners’ surveys.

Urinary tract diseases were documented in 18.7% of the evaluated GPs, higher than the 4.2–5.05% reported in most previous studies ([7], 4.2%); ([14], 5.05%). Urolithiasis specifically (8.3%) also exceeded earlier figures ([9], 5.3%); ([14], 5.05%]). Retrospective case series vary widely, and the literature has no consensus on sex predisposition: some datasets suggest females are affected more frequently [7,9,17]. A 14-year case series reported a general predominance of urolithiasis in males, while cystitis and urinary tract infection mostly affected females [27]. However, Dadah et al. (2025) found that urolithiasis is more common in males [28]. In the present study, sex was not significantly associated with the relative frequency of urinary diseases. Diet plays an important role in developing urinary tract disease, as GPs excrete excess dietary calcium in their urine. Thus, calcium-rich feeds—such as alfalfa—are likely to be predisposing factors in the formation of calcium carbonate stones [2,29,30]. Edell et al. (2022) recorded the diet as reported by the owners [17]. Urinary disease was most likely not associated with husbandry, as GPs had access to a water bottle (67.7%) and only 11.2% were fed alfalfa. This dietary association derives from other studies and could not be tested here, as feeding data were not available; it is offered only as a possible explanation for the urinary disease we observed. Urolithiasis in this species is best regarded as multifactorial, with genetic, metabolic, and dietary factors all playing a role [17,30,31,32].

Ectoparasitic infestation (16.5%) was less frequent than in several studies ([7], 33.3%); ([9], 21.1%); ([19], 22.9%) but higher than the 5.79% reported by O’Neill et al. (2024) [14]. It showed decreasing odds with age, in contrast to Minarikova et al. (2016), who reported such conditions more commonly in <2-year-olds [7]. The owners’ attention must be drawn to the prevention and risks of ectoparasitic diseases, particularly due to zoonotic risks [23].

Reproductive disorders were diagnosed in 16.2% of animals, comparable with the 20% reported by Minarikova et al. (2016) [7], and were dominated by cystic ovarian disease. Ovarian cysts were much more common (32.40% of the intact females) than in other surveys ([7], 21.9%); ([9], 13.7%); ([14], 3.75%); ([19], 2.4%); ([25], 21.9%). This may be because the GPs underwent a veterinary examination. According to the data in the literature, ovarian cysts are widely underdiagnosed, as GPs may be asymptomatic [14]. In a post-mortem study, 37.4% of females were affected [33]. In an ultrasound examination (*n* = 221), 41.6% of GPs had an ovarian cyst. Cyst size and prevalence significantly increased with age [9,34]. Age association was also confirmed in the present study. The present study also confirmed an age association. Because this condition is common, worsens with age, and is preventable, owners should understand the importance of neutering.

The relative frequency of ocular diseases (10.8%) was similar to that reported in other studies ([7], 15%); ([14], 13.7%). Williams and Sullivan (2010) performed ophthalmological examinations on every animal, which can explain the very high 45% prevalence of ocular disorders [16]. Specifically, this included subclinical lens abnormalities (21%), cataract (17%), conjunctivitis (4.7%), keratitis (3.6%), lipid deposition in the conjunctiva (2.3%), and ciliary body heterotopic bone formation (0.8%). The authors concluded that ocular diseases are often underdiagnosed. O’Neill et al. (2024) reported conjunctivitis (4.0%) and corneal ulcer (4.9%), similar to our data (2.0% and 1.7%, respectively) [14]. Due to the high rate of underdiagnosis, it is important to remind owners of the importance of preventive examinations.

The literature reports wide variability in the occurrence of respiratory problems ([7], 4%); ([9], 19.7%); ([14], 9.25%); ([19], 8.5%). We recorded a higher incidence of 10.3%, with rhinitis accounting for 43.5% of the respiratory diseases. This may also reflect a link between rhinitis and dental disease. In comparison, upper respiratory tract symptoms or noises occurred only in 1.5% of cases. Two previous studies reported a higher prevalence of such conditions (9.7%, [9]; 7.6%, [14]). Pneumonia (3.4%) was less common than in O’Neill et al. (2024, 5.07%) [14]. In addition to infectious agents, dusty hay or substrate has been proposed as an environmental contributor to respiratory disease [35]. Because we did not collect husbandry information, we cannot assess whether these factors contributed to the respiratory cases recorded here.

The prevalence of obesity varies across previous studies (2.14–23%) [7,9,14,15]. Obesity is a husbandry-related problem caused by an improper diet (low in fiber and high in energy) and low physical activity, most likely due to the small enclosure. Obesity predisposes to osteoarthritis, subconjunctival lipid deposition or “fatty eye”, and pododermatitis [2,7,8,36]. Calorie restriction, and consequently weight loss, can reduce the severity of osteoarthritic lesions in the knee by 56% in GPs [37,38]. Diets high in fat and energy lead to obesity, glucose intolerance, dyslipidemia, nonalcoholic fatty liver disease, cardiac, and kidney disease in GPs [5,6]. Because the extent of obesity is not always clear to pet owners, it is important to remind them to perform regular weight checks. This also allows the timely detection of weight loss caused by dental or gastrointestinal disease.

Gastrointestinal disorders were observed in 7.3% of cases, in accordance with Minarikova et al. (2016, 13.1%) [7]. It was primarily associated with gastrointestinal stasis, a life-threatening condition caused by the accumulation of gas, dilatation of the stomach and caecum [39,40]. Jekl et al. (2008) reported a significantly higher occurrence of meteorism, liquid gut contents, and diarrhea compared with rabbits [24]. In small herbivorous mammals, gastrointestinal problems are commonly associated with dental disorders [7,41]. Hay, as a fibrous feedstuff, is the most important component of the GP’s diet to maintain gastrointestinal health [1,42,43,44]. Also, a diet high in fruits increases the risk of gastrointestinal problems in herbivorous small mammals [39,45,46]. Because data are limited, we could not establish a similar correlation.

The age pattern of fungal diseases is consistent with an acquisition-related epidemiology, in which younger GPs are the principal reservoir [14,47]. Among the fungal diseases, dermatophytosis is the most common [14] and remains widely underdiagnosed [23], so the low recorded frequency should not be taken as reassuring. d’Ovidio et al. (2014) examined asymptomatic GPs, and fungal growth was present in 20% [23]. Owners should be advised accordingly, and comparable zoonotic caution applies to ectoparasitic disease.

The long asymptomatic phase may also contribute to the lower incidence of liver disease (4.2%). Traumatic injuries (3.5%) are mostly associated with a lack of caution during handling or fighting and were reported at a similar frequency ([9], 2.1%); ([14], 5.8%). Knowledge of proper care requirements and how to handle animals correctly can prevent most of these injuries.

The low incidence of pododermatitis (3.3%) indirectly indicates good hygiene and housing conditions of GPs. Based on the literature, pododermatitis is among the least common diseases ([7], 3.0%); ([9], 2.5%); ([14], 1.4%). One exception is a study that examined the prevalence of skin diseases and found that nearly half of the GPs were affected [8]. Educating pet owners about proper care conditions, such as access to different surfaces, can further reduce the incidence of pododermatitis [48].

The relative frequency of skeletal and joint diseases was consistent with other studies and occurred rarely [7,14]. Skeletal diseases may indicate nutritional deficiencies; therefore, nutritional guidance should always be part of the routine clinical examination. A similar pattern applies to cardiovascular disease, showing low relative frequencies in this and previous studies ([7], 1.2%); ([49], 2.8%). Because the mean and median age of GPs with cardiovascular disease was the highest, preventive cardiac examination may benefit older GPs.

The relative frequency of thyroid diseases (1.9%) may reflect underdiagnosis ([7], 0.4%); ([50], 1.9%). Previous surveys mostly reported tumors, while we only recorded one case of thyroid tumor [7,51,52,53]. Unspecific clinical signs, such as weight loss and poor coat quality, may raise concern for hyperthyroidism. Overgrown nails are a relatively common husbandry-related condition ([9], 11.6%); ([14], 26.5%). However, it was much less common in this cohort (0.7%). This indicates good housing conditions, as access to different surfaces helps ensure proper claw wear [48]. Here again, owner education is key. Hypovitaminosis C (scurvy) is frequently subclinical, which may explain why no cases were diagnosed in this cohort. The low incidence of pododermatitis (3.3%) may indirectly indicate good hygiene and housing conditions. Nevertheless, this is speculative, as no husbandry data were available.

## 5. Conclusions

A large proportion of the GPs presented to the clinic had one or more disorders. Although nutrition, husbandry, genetics, and age are recognized contributors to many of these conditions in the literature, our dataset did not include dietary or housing information (a limitation of the study), so we cannot attribute the observed frequencies to these factors. However, veterinarians should be made aware of the importance of educating pet owners. For example, spaying can prevent ovarian cysts, which occur at a high rate in females. Regular, detailed dental examinations of GPs are important for both prevention and early diagnosis. Age was associated with the recorded incidence of several diseases; however, diagnostic capabilities varied among animals and were generally higher in older individuals. After accounting for the number of examinations, age remained independently associated with ovarian cysts, neoplasms (tumors) and tumor-like lesions, gastrointestinal, hepatobiliary, and urinary disease. Thus, the odds of these conditions increased significantly with age, independently of the number of veterinary visits. In contrast, after adjustment, age was no longer associated with dental or respiratory diseases. Accordingly, these findings may reflect differences in diagnostic opportunity. Regardless, regular veterinary check-ups for GPs ≥ 3 years of age are recommended. The inverse associations for ectoparasites and fungal disease persisted. Thus, owners should also be made aware of ectoparasites and fungal infections.

The study population consisted exclusively of GPs presented to a referral exotic veterinary clinic. Consequently, disease frequencies cannot be interpreted as population prevalence. The prevalence presented here should therefore be interpreted primarily as the cumulative proportion of animals in which the various conditions were recorded at a single veterinary practice over a variable observation period, rather than as incidence or population prevalence. A more precise understanding of the causes of these diseases requires studies in which veterinarians examine GPs and detailed information is available on their housing and feeding conditions.

## Figures and Tables

**Figure 1:**
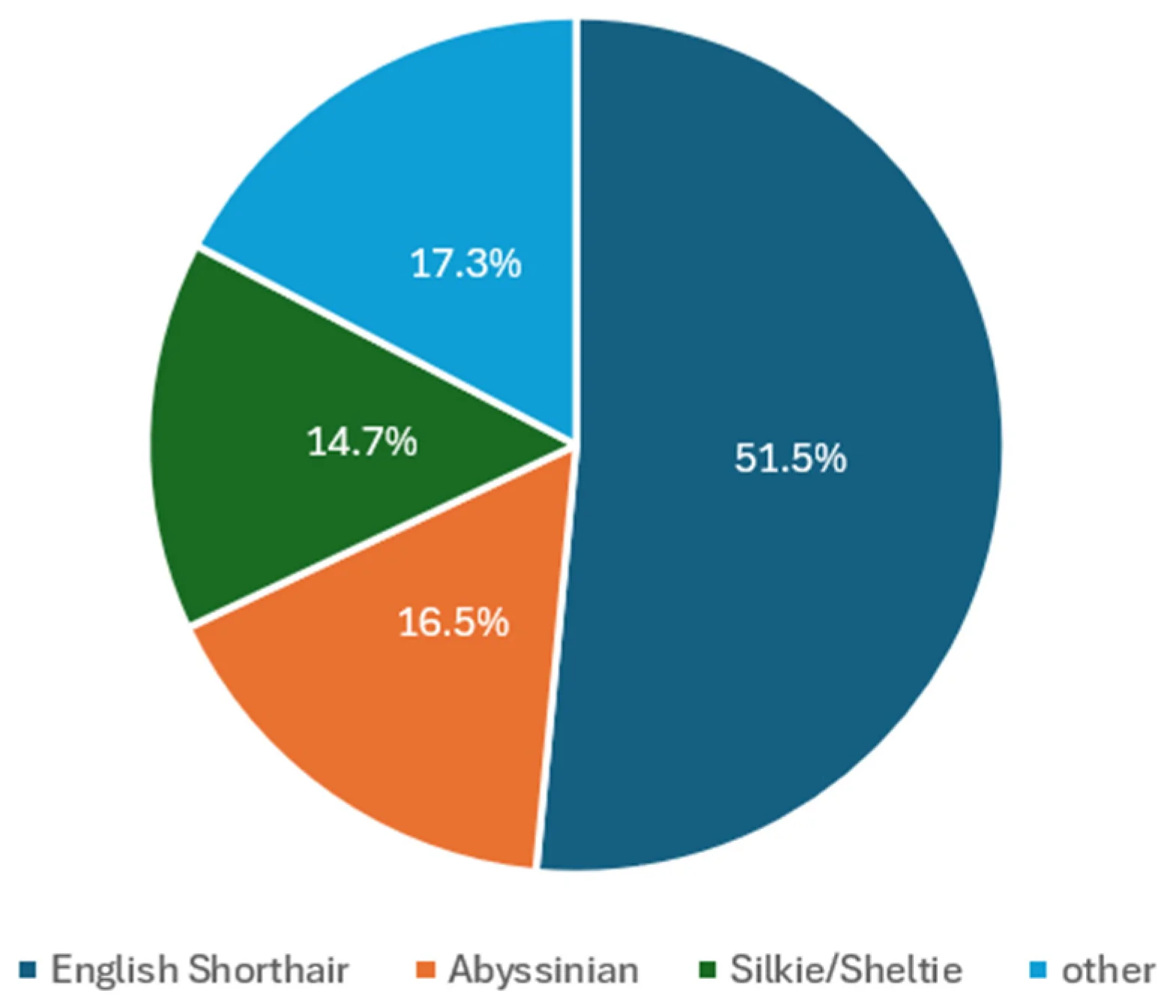
The registered Guinea pig breeds. Other: English Crested (4.7%, *n* = 28), Baldwin (3.8%, *n* = 23), Peruvian (2.0%, *n* = 12), Rex (2.0%, *n* = 12) and American (1.8%, *n* = 11), American Crested (1.0%, *n* = 6), American Teddy (0.5%, *n* = 3), Swiss Teddy (0.5%, *n* = 3), Texel (0.3%, *n* = 2), Lunkarya (0.2%, *n* = 1), and unknown (0.5%, *n* = 3).

**Figure 2:**
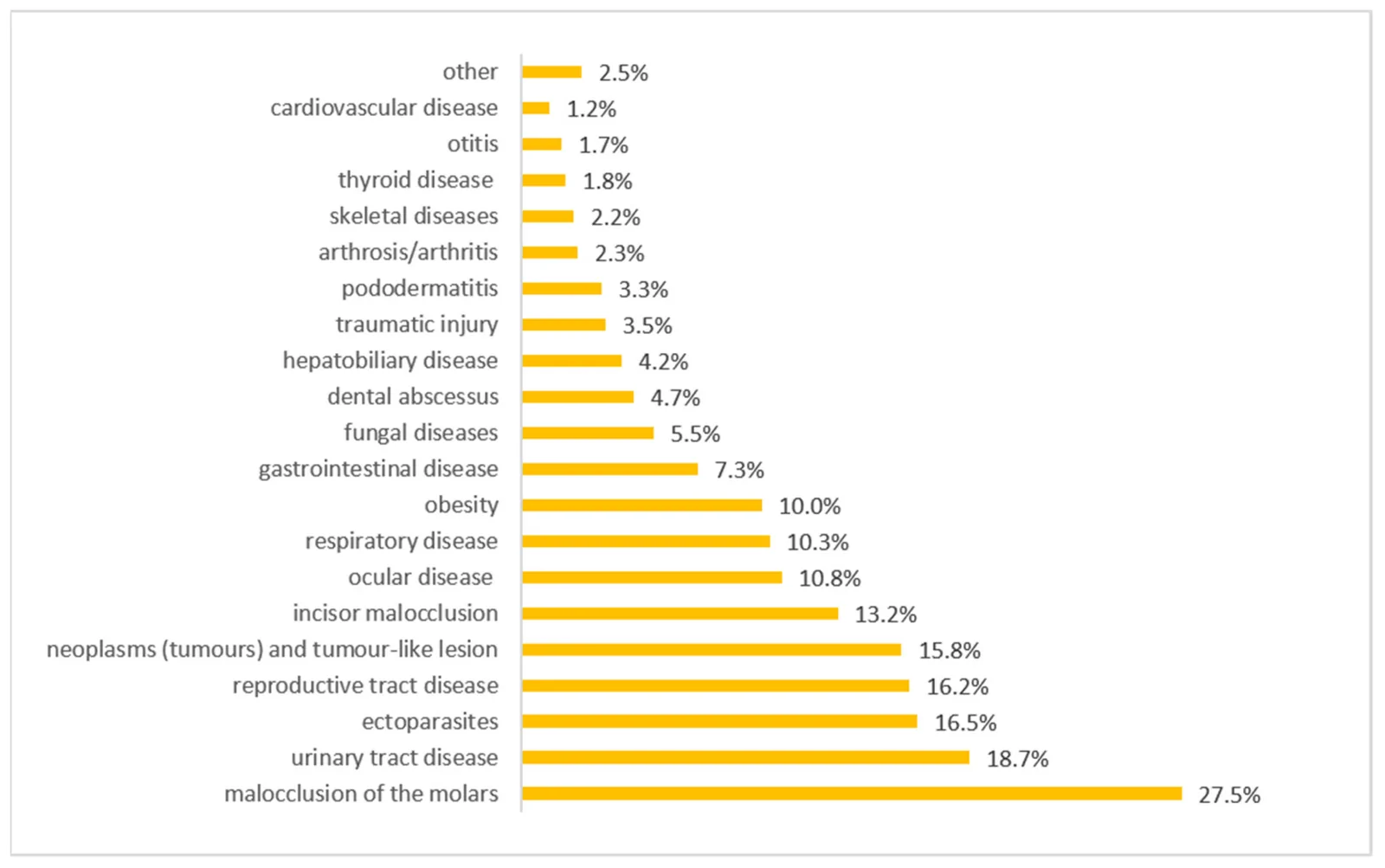
Frequency of diagnosed conditions among the 600 Guinea pigs; percentages are calculated relative to the whole cohort (*n* = 600). Other: overgrown nails (*n* = 4), suture failure (*n* = 3), hernia (*n* = 3), toxicosis (*n* = 3), and coagulopathy (*n* = 2).

**Figure 3:**
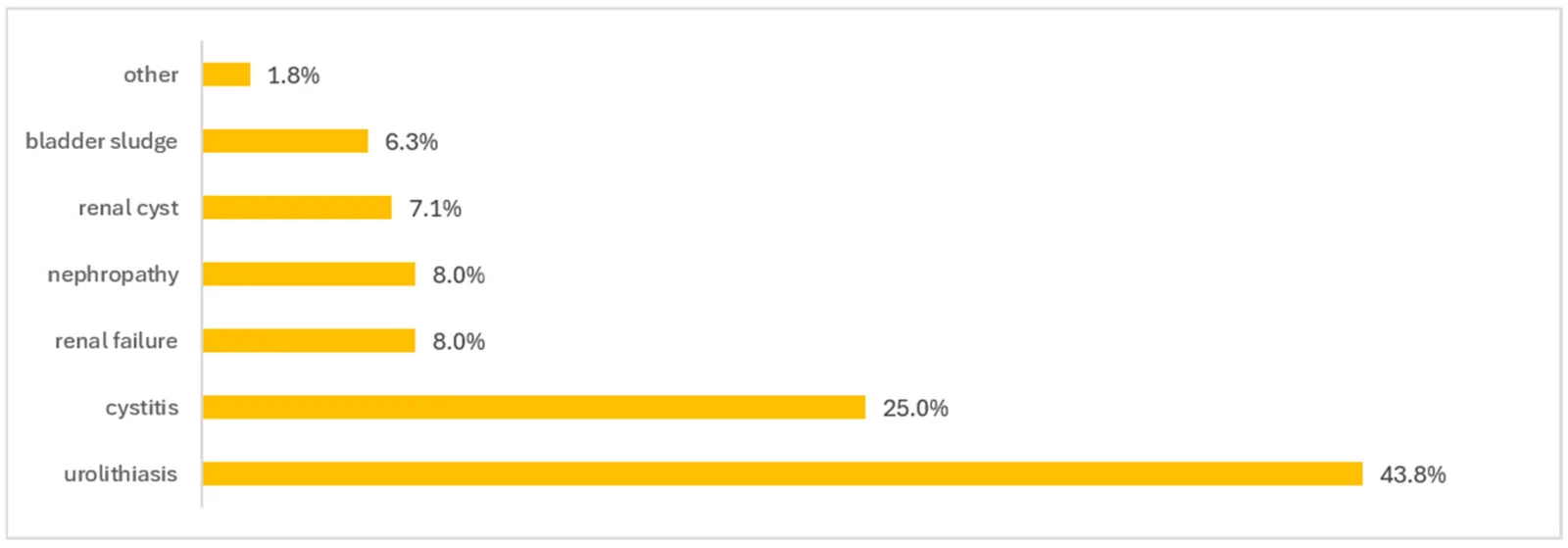
Distribution of urinary-tract diagnoses among the 112 Guinea pigs with urinary-tract disease; percentages are calculated relative to affected animals (*n* = 112), not to the whole cohort. Other: hydronephrosis and nephrolithiasis.

**Figure 4:**
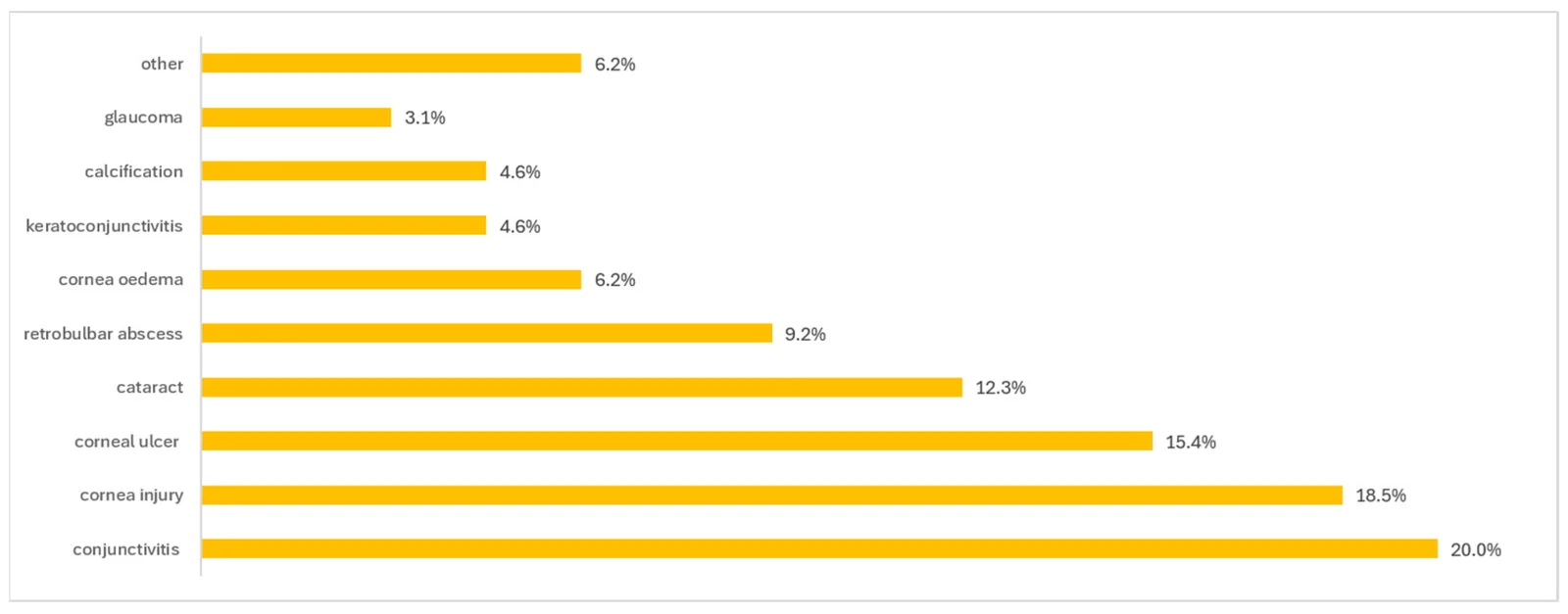
Distribution of ocular diagnoses among the 65 Guinea pigs with ocular disease; percentages are calculated relative to affected animals (*n* = 65), not to the whole cohort. Other: panophthalmitis, microphthalmos, iridocyclitis, and right-sided lens dislocation.

**Figure 5:**
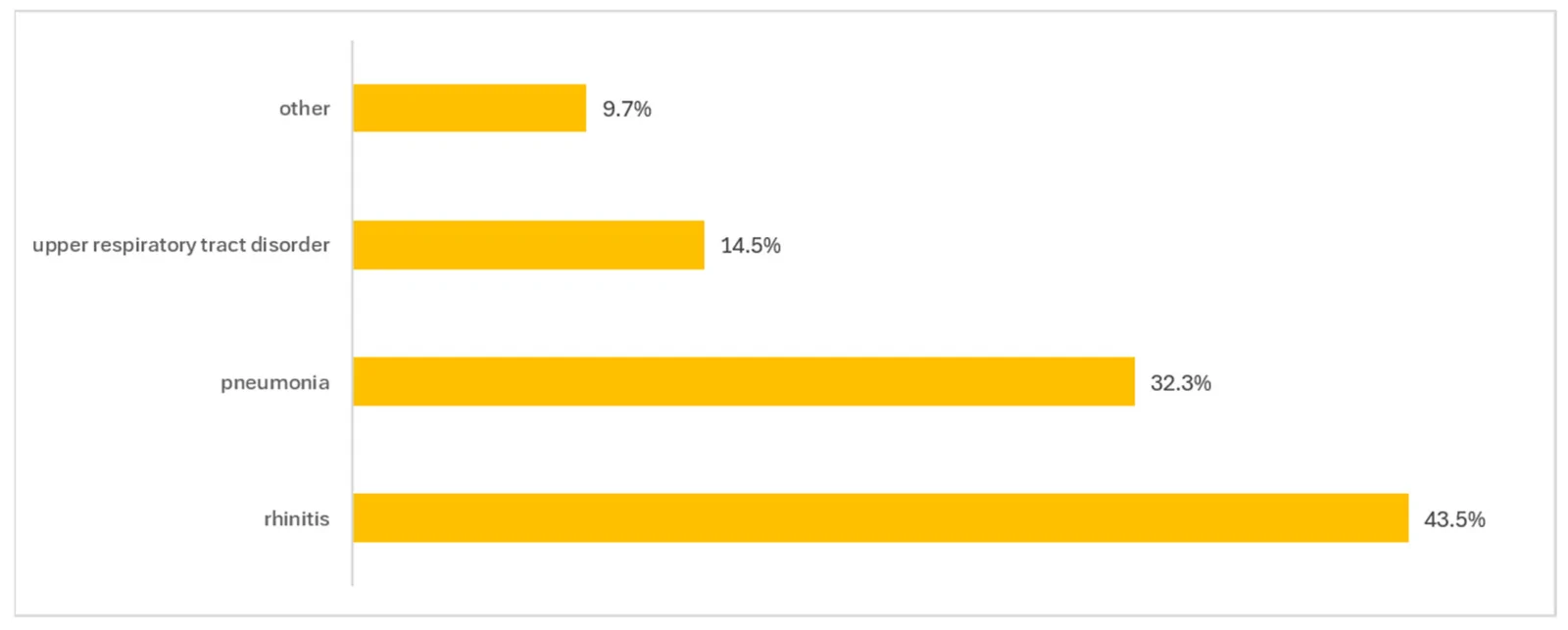
Distribution of respiratory diagnoses among the 62 Guinea pigs with respiratory disease; percentages are calculated relative to affected animals (*n* = 62), not to the whole cohort. Other: bronchitis, rhinoconjunctivitis, aspiration, pharyngitis, and pulmonary oedema.

**Figure 6:**
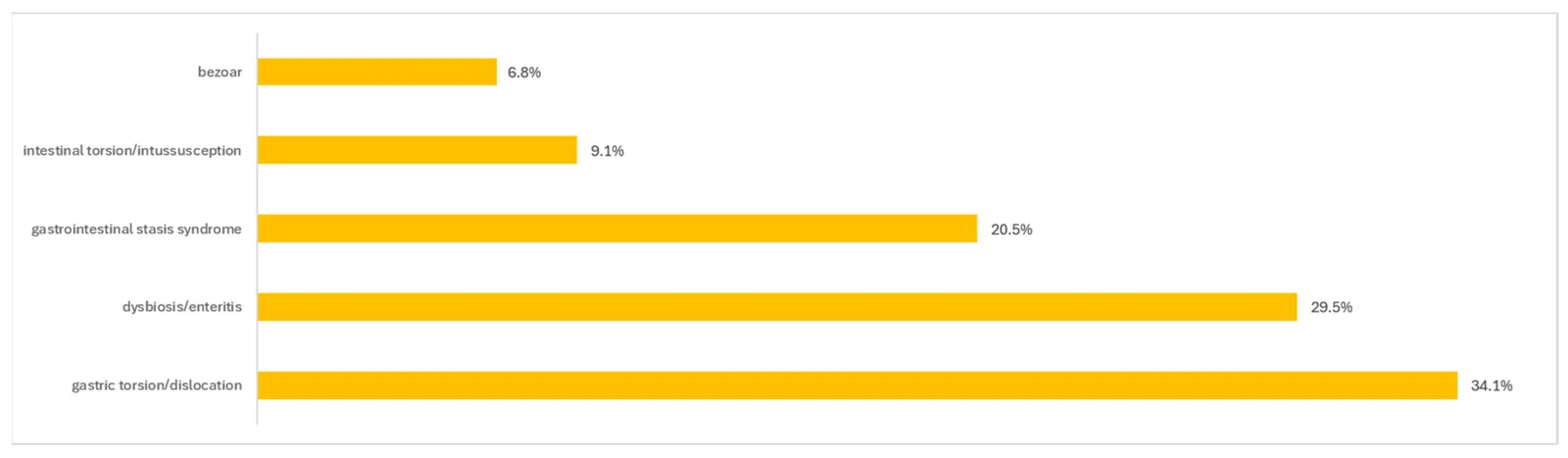
Distribution of diagnoses associated with gastrointestinal diseases among the 44 Guinea pigs with gastrointestinal disease; percentages are calculated relative to affected animals (*n* = 44), not to the whole cohort.

**Figure 7:**
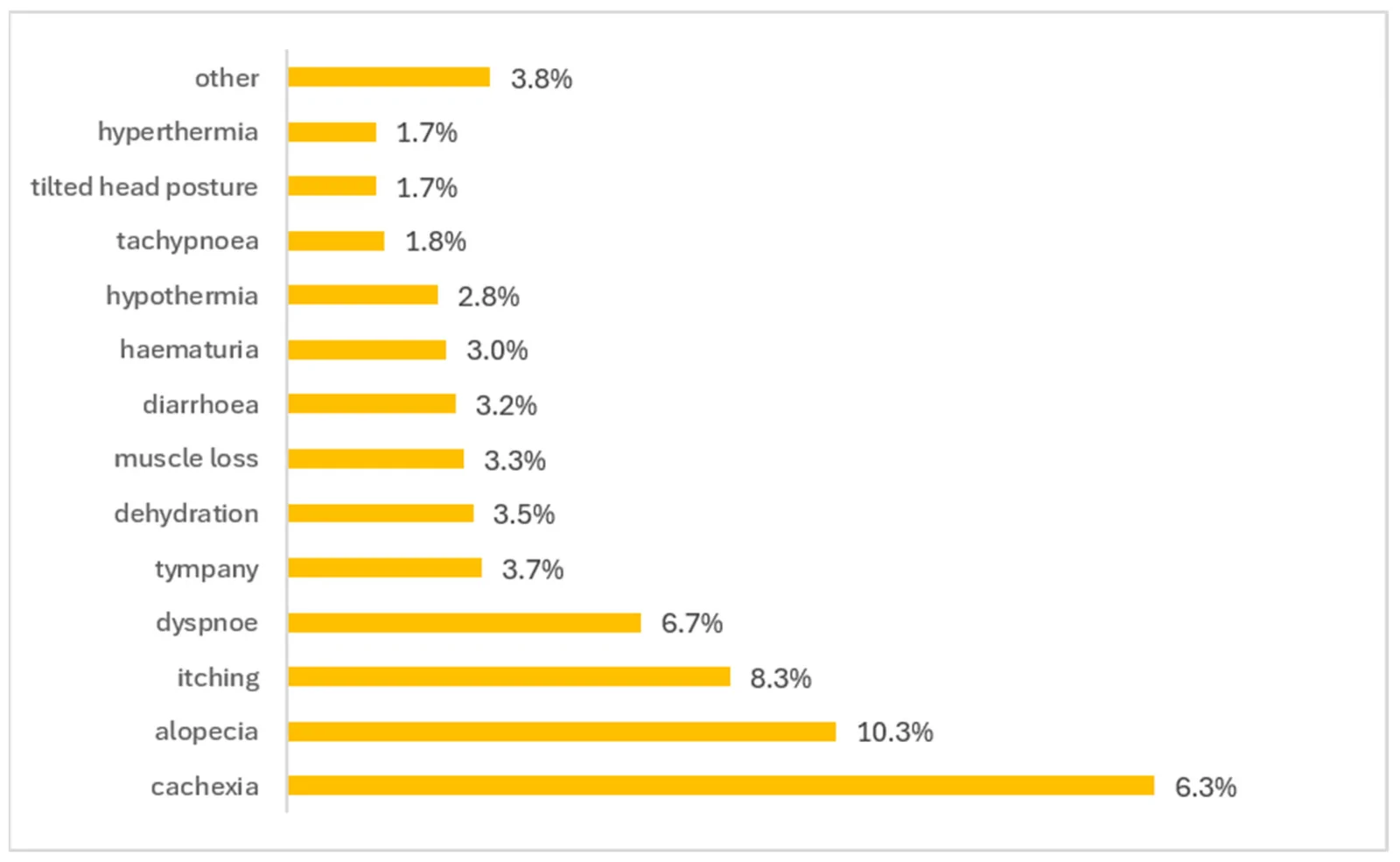
The most frequently recorded clinical signs among the 600 Guinea pigs; percentages are calculated relative to the whole cohort (*n* = 600). Other: cardiac symptoms (*n* = 9), exophthalmos (*n* = 6), neurological symptoms (*n* = 5), and epiphora (*n* = 3).

**Table 1:** Recorded parameters with results.

Age	Mean age of GPs at the last veterinary visit was 38.9 ± 23.2 months, range: 1–107-month-old
Sex	51.3% (*n* = 308) male and 48.7% (*n* = 292) female
Neutering status	Neutering rate: 17.3%, *n* = 104 *n* = 70 male (22.7% of the males), *n* = 34 female (11.6% of the females)
Body weight	Mean: 0.9 ± 0.1 kg
Total number of veterinary visits	Mean: 3.0 ± 3.2 visit/animal, range: 1–26 visit/animal
Number of veterinary treatments for dental diseases	Mean: 2.5 ± 3.3 visit/animal, range: 1–23 visit/animal

**Table 2:** Distribution of molar involvement.

Tooth	Tooth	Number of Affected Teeth
Left maxillary	Premolar	59
	Molar 1	59
	Molar 2	65
	Molar 3	61
Right maxillary	Premolar	72
	Molar 1	76
	Molar 2	78
	Molar 3	79
Left mandibular	Premolar	67
	Molar 1	53
	Molar 2	50
	Molar 3	51
Right mandibular	Premolar	65
	Molar 1	61
	Molar 2	57
	Molar 3	53

**Table 3:** The 95% confidence intervals for the age (in years) of the Guinea pigs affected by that condition.

Disease	Mean-Age [95% CI]	Median-Age [95% CI]
Malocclusion of the molars	3.57 [3.32, 3.82]	3.33 [3.08, 4.00]
Incisor malocclusion	3.69 [3.28, 4.10]	3.33 [3.08, 4.00]
Dental abscess	3.62 [3.17, 4.07]	3.58 [3.08, 4.25]
Obesity	3.15 [2.82, 3.50]	3.12 [2.50, 3.75]
Gastrointestinal disease	4.08 [3.47, 4.69]	3.88 [3.08, 4.25]
Ovarian cysts *	4.28 [3.99, 4.57]	4.25 [3.92, 4.58]
Neoplasms (tumors) and tumor-like lesions	4.25 [3.89, 4.61]	4.25 [3.83, 4.75]
Respiratory disease	3.75 [3.28, 4.22]	3.92 [2.75, 4.58]
Urinary disease	3.89 [3.55, 4.22]	3.75 [3.33, 4.08]
Fungal disease	2.16 [1.50, 2.81]	1.75 [0.67, 2.92]
Ectoparasites	2.55 [2.16, 2.93]	2.00 [1.67, 2.33]
Ocular disease	3.66 [3.17, 4.15]	3.92 [2.50, 8.17]
Hepatobiliary disease	4.10 [3.55–4.85]	3.75 [2.92–5.00]
Arthritis/arthrosis	4.17 [2.85–5.48]	3.42 [2.17–8.17]
Cardiovascular disease	5.56 [3.61–7.51]	5.67 [2.50–8.17]

CI = confidence interval, * only females.

**Table 4:** The adjusted odds ratios with 95% confidence intervals for 13 diseases.

Disease	Coat (Long) OR [95% CI]	Sex (Male) OR [95% CI]	Age (per yr) OR [95% CI], Unadjusted	Age (per yr) OR [95% CI], Adjusted
Malocclusion of the molars	0.76 [0.53, 1.15]	1.72 [1.20, 2.48] *	1.14 [1.04, 1.25] *	1.10 [0.99, 1.22]
Incisor malocclusion	0.62 [0.35, 1.06]	2.15 [1.32, 3.57] *	1.17 [1.03, 1.33] *	1.12 [0.97, 1.29]
Dental abscess	0.68 [0.27, 1.56]	1.0 [0.47, 2.16]	1.11 [0.91, 1.36]	1.08 [0.86, 1.34]
Obesity	1.43 [0.82, 2.44]	0.78 [0.45, 1.33]	0.96 [0.83, 1.11]	0.95 [0.82, 1.11]
Gastrointestinal disease	1.48 [0.78, 2.66]	1.57 [0.84, 2.99]	1.29 [1.10, 1.51] *	1.29 [1.09, 1.52] *
Ovarian cysts	0.93 [0.54, 1.58]	NA	1.43 [1.25, 1.65] *	1.51 [1.28, 1.77] *
Neoplasms (tumors) and tumor-like lesions	1.12 [0.69, 1.77]	0.81 [0.51, 1.26]	1.38 [1.23, 1.56] *	1.43 [1.27, 1.62] *
Respiratory disease	0.68 [0.61, 1.85]	1.26 [0.75, 2.16]	1.17 [1.02, 1.34] *	1.16 [1.00, 1.34]
Urinary disease	0.66 [0.42, 1.06]	0.90 [0.60, 1.37]	1.24 [1.11, 1.38] *	1.23 [1.09, 1.38] *
Fungal disease	1.08 [0.50, 2.24]	0.43 [0.20, 0.89]	0.66 [0.52, 0.83]	0.70 [0.55, 0.88] *
Ectoparasites	0.90 [0.56, 1.43]	0.85 [0.55, 1.31]	0.76 [0.67, 0.87] *	0.74 [0.65, 0.86] *
Ocular disease	1.66 [0.98, 2.80]	0.85 [0.51, 1.43]	1.13 [0.99, 1.29]	1.12 [0.97, 1.29]
Hepatobiliary disease	0.97 [0.39, 2.22]	1.61 [0.71, 3.77]	1.28 [1.04, 1.58] *	1.25 (1.01–1.56) *

OR = odds ratio, CI = confidence interval, NA = not available, * *p* < 0.05.

## Data Availability

Data owned by the veterinary practice can only be shared with the owner’s consent. Residual re-identification risk within this single-center cohort requires controlled access. Anonymized data will be shared with qualified researchers for legitimate academic purposes.

## Supplementary Materials

The following supporting information can be downloaded at: https://www.mdpi.com/article/10.3390/ani16193026/s1, Table S1: Diagnostic criteria and methods of presumptive and confirmatory diagnoses.

## Abbreviations

The following abbreviations are used in this manuscript:

GP	Guinea pig
