# Supplementary material for: Diseases of Pet Guinea Pigs (Cavia porcellus) Presented to a Specialist Exotic-Animal Clinic: A Retrospective Study
Source: Animals (Basel). 2026 Sep 25;16(19):3026. doi: 10.3390/ani16193026 (PMC13663525; doi:10.3390/ani16193026)
Supplement: Supplementary file 1 [file animals-16-03026-s001.zip › animals-4465699-supplementary.pdf]

Supplementary Table S1. Diagnostic criteria and methods of presumptive and confirmatory diagnoses

Disease category	clinical signs	presumptive diagnoses	confirmatory diagnoses
Digestive system (oral and gastrointestinal)			
Malocclusion of the molars	selective or reduced appetite, quidding, dysphagia, ptyalism, drooling, progressive weight loss, reduced fecal output, gastrointestinal hypomotility, epiphora	history and clinical signs together with limited conscious oral examination and extraoral palpation of apical elongation	oral examination under general anesthesia, additional skull radiography, computer tomography
Incisor malocclusion	overgrown, misaligned or fractured incisors, drooling, anorexia and weight loss.	history and clinical signs together with conscious visual examination of the incisors	oral examination, conscious or under general anesthesia
Dental abscess	facial, mandibular or retrobulbar swelling; exophthalmos (depending on the location of the abscess), anorexia, ptyalism, pain, draining tract (occasionally)	palpable facial/mandibular swelling with concurrent dental disease	radiography or computer tomography demonstrating apical lucency, tooth-root involvement, lysis of the bone tissue or osteomyelitis
Hepatobiliary disease	anorexia, lethargy, weight loss, ptyalism, icterus, hepatomegaly	clinical signs supported by blood biochemistry	ultrasonography, hepatic cytology/histopathology
Urinary system			
Urinary tract disease	pain (vocalization on urination, stranguria, pollakiuria, hunched posture), perineal urine scald, hematuria, dysuria, anorexia, weight loss, palpable bladder or calculi	clinical signs with abdominal palpation and urinalysis findings (hematuria, crystalluria, pyuria).	radiography, ultrasonography; urinalysis, urolith analysis, blood biochemistry
Reproductive system			
Ovarian cyst	weight gain, decreased appetite, lethargy, hunched posture, bilaterally symmetric (non-pruritic alopecia), abdominal distension, palpable mid-abdominal mass, behavioral change	clinical signs	ultrasonography, radiography, surgery

Male and female reproductive tract disorders (excluding ovarian cysts)	Female: lethargy, anorexia, weight loss, vaginal discharge or hemorrhage, dystocia, swollen, painful, discolored mammary glands (mastitis), infertility<br>Male: scrotal/testicular swelling or asymmetry, preputial swelling or discharge, dysuria, penile protrusion/paraphimosis, entrapment of hair or bedding around the penis, infertility	clinical signs	see “presumptive diagnoses” if clinical symptoms define the condition; radiography, ultrasonography, blood biochemistry
Respiratory system			
Respiratory diseases	nasal/ocular discharge, sneezing, dyspnea, tachypnoea, increased effort, open-mouth breathing, abnormal lung sounds, anorexia and lethargy	clinical signs with abnormal thoracic auscultation.	thoracic radiography, nasal bacterial culture if needed
Cardiovascular system			
Cardiovascular disease	dyspnoea, tachypnoea, lethargy, anorexia, exercise intolerance, heart murmur, pleural effusion and pulmonary oedema (congestive failure)	clinical signs with auscultatory abnormality, radiography	see “presumptive diagnoses”, echocardiography (cardiomyopathy)
Ophthalmic system			
Ocular diseases	ocular discharge, epiphora, blepharospasm, conjunctival hyperemia, corneal lacrimal prolapse, cataract	clinical signs	fluorescein (ulcer), Schirmer (keratoconjunctivitis sicca), corneal/conjunctival cytology and culture (infectious keratitis/conjunctivitis), radiography, computer tomography or ultrasonography (retrobulbar disease, heterotopic bone)
Aural system			
Otitis	tilted head, ataxia, nystagmus, aural discharge, head shaking	clinical signs with otoscopic findings	radiography, computer tomography, cytology and culture of aural exudate
Integumentary system (non-infectious)			

Pododermatitis	swelling, erythema, hyperkeratosis, ulceration and crusting of plantar/palmar surfaces of the foot, lameness and pain, deep infection, tendon involvement and osteomyelitis (in chronic cases)	clinical findings, characteristic foot lesions	see "presumptive diagnoses", no additional test is needed; radiography can be used to assess osteomyelitis in advanced cases
Overgrown nails	excessively long, curled claws, altered gait	visual assessment of claw length	see "presumptive diagnoses", no additional test is needed
Infectious and parasitic disease			
Fungal disease	circular patches of alopecia (often typical localization, ringworm), scaling, crusting, erythema, pruritus	clinical signs, typical lesion distribution, microscopy, Wood's Lamp (low sensitivity in ringworm)	microscopy of hairs/scale, fungal culture
Ectoparasites	pruritus, alopecia, excoriation, scaling, visible parasites	clinical signs and characteristic distribution	microscopy of skin scrapings, tape/brushing preparations
Neoplasms (tumors) and tumor-like lesions			
Neoplasms (tumors) and tumor-like lesions	cutaneous/subcutaneous masses, mammary masses, respiratory signs (pulmonary/thoracic masses), abdominal mass, progressive weight loss, cachexia.	palpable or imaged mass	cytology, histopathology, radiography, ultrasonography, computer tomography
Gastrointestinal disease	anorexia, lethargy, hunched posture, abnormal hard faces (change in shape and size, reduced amount, absent, diarrhea), gastric and/or cecal tympany, gastric dilatation, dehydration, weight loss	clinical signs, abnormal borborygmi	radiography (contrast can be used if needed), ultrasonography, complete blood count and a biochemistry
Musculoskeletal system			
Arthritis/arthrosis	lameness, stiffness, reluctance to move, joint swelling, pain and reduced activity, impaired grooming	clinical signs with localizing joint findings	radiography, arthrocentesis with synovial-fluid analysis (to exclude septic arthritis)
Skeletal diseases	bone deformity (spinal deformity), pain, reluctance to move	clinical and dietary findings (calcium, phosphorus, vitamin D, vitamin C)	radiography, blood biochemistry

Endocrine and metabolic disease			
Thyroid disease (hyperthyroidism)	weight loss despite a normal or increased appetite, hyperactivity, tachycardia, cervical mass, poor coat, polyuria/polydipsia	clinical signs with a palpable cervical mass	see "presumptive diagnoses"; elevated serum total thyroxine with and without elevated free T4 supports the diagnosis; ultrasonography, histopathology (to define tumor type)
Obesity	excess body mass; impaired self-grooming, reduced mobility	elevated body weight relative to breed and age; body condition scoring	see "presumptive diagnoses", no additional test is needed
Trauma			
Traumatic injury	lameness, non-weight-bearing, swelling, wounds, pain, limb deformity, hemorrhage, neurological deficits with spinal injury	history and clinical findings	see "presumptive diagnoses"; radiography, computer tomography (spinal/complex injuries) if needed
Other diseases			
Hernia	reducible or non-reducible swelling at the body wall of umbilicus or inguinal region; dyspnoea and gastrointestinal signs (diaphragmatic hernia), pain and obstruction in incarcerated hernia	clinical signs with characteristic swelling	radiography, ultrasonography
Coagulopathy	petechiae and ecchymoses, prolonged bleeding, epistaxis, hematoma, pallor and weakness	clinical bleeding signs with a compatible history (diet, toxin exposure, sepsis)	coagulation profile (PT/aPTT), platelet count, complete blood count; response to ascorbate supplementation (vitamin C deficiency)
Toxicoses	toxin-dependent clinical signs, gastrointestinal and neurological signs are common	history of exposure with compatible clinical signs	documented exposure, specific toxicological assays on blood and/or tissue (post-mortem)
